# Systematic Analysis of Literature on the Healthcare Financial Models to Follow in Russia and Romania

**DOI:** 10.3390/healthcare10061086

**Published:** 2022-06-10

**Authors:** Vladimir Bulatnikov, Cristinel Petrişor Constantin

**Affiliations:** Faculty of Economic Sciences and Business Administration, Transilvania University of Brasov, 500068 Brasov, Romania; vladimir.bulatnikov@unitbv.ro

**Keywords:** healthcare models, health insurance model, private health model, public health model, mixed health model, financing healthcare systems

## Abstract

This paper aims at finding the suitable healthcare financial model, focusing on their pros and cons, as debated by several scholars. The focus is on the potential benefits for both Romanian and Russian healthcare systems. To reach this goal, a systematic review of the literature was conducted, and various competitive advantages and disadvantages of the financial models were extracted. We reviewed 77 papers published during the last 21 years that were found in famous scientific databases. The main findings of the research point out that the financing of healthcare systems should be based on hybrid sources, and the funds raised should be better invested in order to create added value. By assuring a proper financing, the population’s quality of life will improve and life expectancy will increase. This paper provides a new viewpoint to the problem because it reviews certain papers from Russian literature which are not usually included in the review articles. The research results have implications for the government, medical community, and academia, which should work together to strengthen the healthcare system.

## 1. Introduction

The development of a modern healthcare system is a critical step in attaining a country’s strategic economic growth goals [1]. Contemporary methodological techniques for evaluating the efficacy of healthcare activities based on the study of their essential components must be developed and applied. Thus, the findings of various scholars published in the specialty literature might provide scientific background for managerial choices in the development of healthcare systems [2]. The management of healthcare systems encompasses a wide variety of tasks, such as research, planning, execution, and control, all of which are largely focused on the development, advancement, and assessment of results in order to achieve a healthcare organization’s goals [3]. It is essential to address the issue of conserving human health and extending the active lifespan of humans. Thus, measures such as the availability of medical care, the quality of medical services supplied, and the attainment of important industrial development indicators can assure the success of the healthcare system. The development and application of modernizations in healthcare, both in terms of disease prevention and treatment, as well as in terms of medical institutional socioeconomic development, is particularly important for the sustainable development of the society and is one of mankind’s priority areas, alongside energy efficiency and energy conservation, nuclear, space, and information technologies [4]. However, all these actions need funds, so the establishment of a proper financing model is crucial [1].

### The Purpose of the Study

There are many review articles in the fields of healthcare financial models (e.g., [5,6]), being progressively more popular and attracting great attention on the private sector, which is growing due to its several benefits to the patients of post-communist countries. In the papers, researchers focus mostly on topics such as public–private partnership [7,8] and private health expenditures [9,10]. The significance of this particular article consists in the review of certain papers from Russian literature which are not usually included in the English review articles. Especially when we consider the comparison with EU countries, these mixed results of the Russian and Romanian experience generate a similar trend of the financial models used in the healthcare system. The logic of choosing the comparison between Russia and Romania consists in similar indicators of healthcare systems and related roots, starting from the Semashko model, even if the countries belong to completely different international entities. This comparison represents the main innovation brought by this study in the field.

Taking into account the above-mentioned, the goal of this paper is to find the suitable healthcare financial model, focusing on pros and cons debated in the literature. To reach this goal, 77 articles selected from scientific databases were reviewed. We intended to provide answers to the following two research questions: Q1. Which financial model is better to follow for the healthcare system? Q2. How to attract, accumulate, and guide payments more effectively through healthcare system? The answers to these questions could help countries like Russia and Romania to improve their healthcare systems by assuring a better financing, taking into account that these countries have many common issues in the history and organization of their healthcare systems [5,6,7].

The article is structured in four sections. The first section contains an introduction to the field, followed by the research methodology. The research results are presented and discussed in Section 3. Section 4 contains conclusions, implications, and further research directions.

## 2. Materials and Methods

A literature review was conducted, and the various competitive advantages and disadvantages of the financing models that require attention from the managers of the healthcare systems were found. We examined more than 80 papers published during the last 18 years found on scientific databases. Finally, the article includes 77 references directly related to the financing of healthcare systems.

### 2.1. Search Strategy

The publications were located in PubMed, Scopus, Science Direct, and the Russian scientific database e-Library. The complete versions of several publications that were not open access were located on social platforms such as ResearchGate, Journal of Medicine and Life, Europe PMC, SPB lib, IDEAS, and others. We utilized the words “Healthcare Model”, “Medical Finance Model”, and “Healthcare System” in all databases. As a discipline, we picked “Business and Management”. Furthermore, we only included articles published in English or Russian in the previous 17 years. Both sides have been observed for domestic sources of information in their native languages—Russian and Romanian—that were translated into the English language so that they could be used in this paper.

### 2.2. Distribution of the Selected Studies

Using the above search criteria in the considered databases, a huge number of records were found. It counted 3011 articles that corresponded to the selected criteria. Using the PRISMA methodology, a set of inclusion and exclusion criteria was used (Table 1), where afterwards 439 articles were selected to be potentially suitable for the study.

Another 385 publications were removed after we checked the entire text of these articles for reasons such as a lack of information on the financial models of the healthcare systems or a lack of information on European countries to conduct a parallel analysis. Articles that lacked information regarding the countries or areas in which they were used were also disqualified.

Finally, 77 papers were included in our systematic review. In the PRISMA Flow Diagram (Appendix A), which was made for a better view of taken steps, the number of included and excluded articles can be found for every step. The studies that were chosen all matched the criteria for inclusion.

### 2.3. Risk of Bias in Included Studies

Since the PRISMA technique needs a risk assessment, the Cochrane risk-of-bias method [11] was used to assess bias in the included papers. It helps to avoid any doubts about the veracity of the findings and contains important information. Random sequence creation, allocation concealment, and blinding of participants are some of the criteria used. Other causes of bias include participants and employees, insufficient outcome data, selective outcome reporting, and other factors. In this case, every article included in this review was assessed according to the risk of bias by analyzing the content of the results, the soundness of the research, and the conclusions, as well as the limitations of the research. The authors rated the articles based on the following grades: unclear, low risk, and high risk. For this analysis, the overall degree of risk was deemed moderate because the purpose was not to conduct an experimental study but to collect a diversity of viewpoints on the financing of the healthcare systems. We did not identify articles with high risk, as they were filtered in the selection process based on the inclusion and exclusion criteria. The journals in which the articles are published are also considered reliable because they are indexed in famous databases.

## 3. Results and Discussions

### 3.1. Q1. Which Financial Model Is Better to Follow for Healthcare System?

There are four main types of organization and financing of the health system in the world. The first type is the presence of a private healthcare model in a market economy like in the USA. “Medical care is provided mainly on a paid basis” [12]. There are several advantages to this system: it urges the physician to perform longer shifts [13], to perform greater responsibilities for the patient, and to visit him more regularly [14], as well as fewer hospital referrals [15]. The major con of this financial system more often is split in two categories: “Disadvantages to Patient Finances” and “Disadvantages to Patient Health” [16]. First category’s flaw is when physicians tend to order more consultations [17], elective procedures [18,19], hospitalizations [20,21], and medical tests [16]. The second category flaw: as a result of the increased patient load, doctors have less time to spend with each patient [22]. Furthermore, the doctor has fewer incentives to offer those patients with well-coordinated care [13]. Duplication of services and/or over-involvement of several physicians may result from a lack of well-coordinated treatment [23]. The second financial model to consider is a “public health model within a market economy” such as Great Britain, Ireland, Denmark, Portugal, and Sweden [24]. One of the cons, for instance, in the Swedish high standard of living and social guarantees for the majority of the population is characterized “by an extensive state social sphere and a high level of budget centralization of the national product: about 60%”. The Swedish healthcare system is very effective, with moderate investment and controlled costs [25]. The third is “the state health model within the state economy”, which existed in former communist countries based on the Semashko healthcare model [26]. This system does not exist anymore in Europe, but the majority of the named countries have adopted a “the mandatory social health insurance model”. The major flaw of this system is the lack of enough financing resources. This shortage is deepened by today’s circumstances of the COVID-19 pandemic crisis, which is leading to a vast decrease in budgetary incomes due to the loss of work for the population and to the growth of public expenditures [27,28]. There is also considerable evidence that apart from the payments included in the statistics, there are some extra-funds paid by patients for healthcare that are deeply integrated into the system in all emerging economies. Numerous publications indicate that these payments provide a significant contribution to the healthcare financing, but this distorts the real costs of healthcare [29,30,31]. The fourth: “A mixed health model in a market economy”, which are found in the most developed countries, such as France, the Netherlands, Austria, Belgium, Switzerland, Canada, Japan [32]. For this type of model, the majority of observed authors say that if both private and public sectors are represented in the medical services market, there will be the economic potential of this market to grow high. Competition between the private and public sectors can become a “managerial stimulus to overcome the inertia and routine in service delivery”, with beneficial effects in adopting innovations [3].

Each country’s healthcare system is considered as a product of its “unique circumstances, history, political life, and national character”. The principles of organization of these systems range from “managed competition” to a rigid structure on the principle of single payer and many intermediate options [33]. If a part of the system is not functioning effectively, it has a negative impact, not only on the system as a whole, but also on its components, which can further “aggravate the situation”. Changing the model, which often require resources, can significantly increase its overall effectiveness [34].

In the following subsections, the main findings regarding the financial models applied in Russia and Romania are presented. If we go down in history, in the last 25 years, the healthcare systems in Russia and Romania have undergone substantial transformations. The most important is the transition to compulsory healthcare insurance, as both countries adopted a mandatory social health insurance model since 1992 and 1997, respectively [35,36]. In addition, there have been at least two crises in the last 13 years that have affected Russia and Romania together [37]. These have served as an incentive for the involvement of market regulators in the work of medical organizations, which has led to a “significant increase in their prospects of attracting additional sources of funding through the provision of paid medical services” [38]. This includes a complex relationship between different organizations created over time and the participants involved the system [39]. It is made up of two levels: national and regional. On the national level, its mission is to achieve government health objectives. On the district level, its mission is to maintain the supervision according to the Ministry of Health’s rules [40].

#### 3.1.1. Coordinates of the Financial Model Existent in Russia

Since the development of healthcare in Russia follows a unique path, it cannot be applied in its pure form as a borrowed foreign national model of healthcare financing [33]. At least because it issues the problem of nonformal payment for health services, which are widely used and characterized by a modest range of institutional forms [41]. Of course, Russian healthcare has fully experienced the results of the systemic crisis of the 1990s, and the attempts to modernize the industry over the past two decades, based largely on foreign experience, have led to a “decrease in the quality and availability, as well as the volume of medical services provided to the population” [42].

The Russian healthcare system has three forms of providing medical care. The first, mandatory healthcare insurance, which replaced budget financing, “covers most of the types of medical care provided to the population” [43]. It was rolled out in 1991 under the strong lobbying influence of market forces: “insurers will compete with each other for the insured and under the so-called competitive model of compulsory health insurance” [39]. The second is paid, carried out at the expense of citizens and enterprises. The ratio of paid and mandatory insurance healthcare aid shows the level of socioeconomic development of the country, according to Russian studies. Here, mandatory insurance should account for more than 90% of the total volume of medical care, and paid care should not exceed 10% [44]. Still, many citizens believe that it will be much better to go to a private clinic than to a municipal one, but not everyone can afford to apply to a paid one, so they choose a free institution [45]. The third one is the fully budgetary finance of public health institutions. In reality, it is more of a supplementary nature, so that it can help the weakest public clinics to struggle with periodical shortages, and for some mostly long-distance clinics, it is the only source of getting periodic flows of money, even though this type of financing can be applicable without insurance financing in the whole country in some particular circumstances [46].

Several positions of the authors regarding the above-mentioned models of healthcare system financing in Russia are presented in Table 2.

The budgetary model of healthcare financing is delightful for Russian reality. “It assumes collection of taxes generated by the revenue and expenditure parts of the system’s budget healthcare”. It will give social priority, focus on prevention, and there will be a highly qualified staff [46].

Social-insurance in Russia has its merits, the essence of which is that “the supplementary medical insurance program, insurance fee on the part of the insured” can lead to an increased quality of healthcare implemented by competing insurers. [39]. First, the existing ways of using the funds accumulated in this system are functionally heterogeneous: “some implement the functions of insurance, some-social assistance, and some a combination of these functions” [43]. Secondly, a large share of costs financed by society and the state are generated by the maintenance of disabled citizens [47]. Here, as well, budgets at all levels are ultimately filled mainly by working citizens, and “it does not encourage working citizens and enterprises to perform high-performance work in any way” [48]. All individuals in the insurance health market are identical in all parameters [49]. Finally, the financial stability of the Russian healthcare system depends not only on the system of compulsory medical insurance as the source of operating expenses, but also from budget investments, which provide capital expenditures [50]. However, there are other statements about the current model which indicates that the current model does not require changes [51].

The private model is characterized by contributions from the personal funds of citizens, charitable foundations, and other sources. The development of private medicine is a form of social medicine entrepreneurship, the feature of which is both the private and public nature of the services provided. Some scientists consider that medical services can be provided by two parties, which are subjects of the private health sector and subjects of the state sector. Even free social medical services should also be provided by structures of the private health sector, which will receive money for these services from budgetary sources [12].

Synthesizing this, these observations are summarized and grouped together in Table 3.

The Russian government collects funds from all levels: “federal, regional and municipal” [39]. In addition to public sources in financing healthcare are used the personal funds of the population; the funding of employers for the treatment of their employees; the funds allocated to the health sector by nonprofit institutions serving the population, and others [53]. However, the lack of special management technologies and the lack of statistical data do not allow to fully assess the level of development of the Russian healthcare model [1].

#### 3.1.2. Coordinates of the Financial Model Existent in Romania

The situation in Romania slightly differs from the Russian one. Like the different nations from Eastern Europe, the Semashko medical care framework was in force in Romania before 1997. It was characterized by a discriminatory distribution of assets, the absence of reaction to the demands of population, and poor administrative actions [54]. Nowadays, there are five financing methods of the healthcare system available in Romania: “financing from the state budget; financing through social health insurance; financing through private health insurance; financing through direct payments; community financing” [55]. What has taken place in Romania after the introduction of the health insurance system in 1997 was in fact the existence of a hybrid system partially financed from the Health Insurance Fund controlled by the Government and the Ministry of Finance, and partially from private sources [56]. Among the European models, the two options for a change would have been: “the actual Bismarck model”, currently used in Germany, Austria, and France, based on insurance, and the “Beveridge model” in Great Britain, Italy, and Sweden, based on general tax revenues [57]. The chosen model was more convenient to the Romanian reality, a middle way between the two options supported by two sides: the supporters of the free market for the functioning of the healthcare system and the supporters of government planning [58]. In fact, during the period following the 1989 moment, in Romania there were not many trained specialists in the fields of healthcare management or healthcare policies [57].

Returning to healthcare financial models, there are two aspects that must be emphasized: “first, there are many financing sources of healthcare expenses; secondly, none of these methods is ideal and cannot provide a magical solution to solve the severe problems, especially in poor countries” [56]. Regarding Romania’s position in healthcare, the different positions of the authors towards different models of healthcare system financing are presented in Table 4.

The budgetary model is the main source that finances healthcare systems in many countries all around the world. Playing an important role is the amount of tax revenues available to different levels of government and regions, which determine the amount that can be provided to health [54]. Social insurance asks who is in charge of regulating it: nonprofit organizations or private insurance organizations? Moreover, it asks how insurance coverage will be provided: by voluntary insurance or compulsory insurance? [59]. The social insurance system relies on a holistic approach of an individual to assess the reduction of his functional capacity and to determine the level of incapacity for work that entitles him to receive various medical services. It analyses the possibilities of rehabilitation and social reintegration for people suffering from an illness or chronic disability [59]. Here, some categories are exempt from insurance contributions: “unemployed, persons doing military service or in penitentiaries, persons on sickness or maternity leave, persons entitled to social security benefits, children under 18 years, persons aged 18–26 years enrolled in any form of education, family members of an insured person” [60]. The present social insurance model in the Romanian healthcare system in some cases has some add-ons from other above-mentioned models [40]. The private model is considered by the authors that has multiple benefits, such as: it gave rise to a competitive market in the medical field; it imposed a permanent increase of the Medical Act quality through performance equipment and well-trained professional personnel; it changed the mentality concerning the relation of doctor–patient; it led to higher levels of health education of the population; it allowed the creation of new jobs, etc., [61]. Concerning the direct payments, the authors consider that the transparency of payments should be assured [40] and the donors model should be well promoted in order to attract funds that indirectly support others who benefit from the healthcare system more than the value of their contributions [62]. A synthesis of the scholars’ findings regarding these financing models is presented in Table 5.

Finally, most of these financing models provide resources that are transferred from healthy people to sick people, so that healthy people contribute more with less benefit from the services and cover the cost of other patients [63]. In spite of the government’s efforts to increase the resources available, such as raising taxes on cigarettes and pharmaceutical companies, the system is still financed largely from compulsory social health insurance and state budget allocation [40].

### 3.2. Q2. How to Attract, Accumulate and Guide Payments More Effectively through Healthcare System?

A significant question about funding healthcare is about how to ensure the funds needed to provide effective medical services. There are five elements available that set the final recipe for financing healthcare systems: “equity, the ability to generate persistent income, efficiency, service quality, and sustainability”. They form the basis of any healthcare budget [64].

Firstly, the problem of insufficient financing of the healthcare system is supposed to be partially solved with public–private partnership [64]. According to scholars, the implementation of public–private partnership in healthcare will: “attract additional financial resources, allow optimizing budget; allow applying the latest scientific and technical developments; improve the quality; increase the availability of medical services” [12]. Under the new model of interaction between the state and business, the main task is to move the state from the “state-pharmacist” to the “state-employee”, where citizens are at the center of the system and play a more active and responsible role than they have been assigned so far [65]. In the meantime, in order to ensure the successful and effective activities, the government bodies should be constantly monitored [66]. Here, public health organizations should compete for the opportunity to attract private investment in order to improve the quality of medical services provided. It is also possible to develop joint brands between private businesses and public healthcare organizations [67]. Public–private partnerships can also have a positive impact on the development of the healthcare providers by fostering innovations, which allow creating flexible and innovative management structures [68].

Secondly, the problem of insufficient attractiveness of some public organizations for insurance companies can be partially solved by monitoring the customer satisfaction and implementing actions to increase this satisfaction: “Customer satisfaction has a positive effect on customer loyalty, which will increase the return on investment” [69]. This evaluation results from the consumption experience [70]. Some authors suggest that patient satisfaction and perceived service quality is essential for the sustainability of healthcare [71]. Communication is also important in establishing and maintaining a steady relationship with the patients, to create a positive attitude, and to stimulate consumers to apply for the healthcare services provided by an organization [72]. It is necessary to restore trust and mutual respect between doctors and patients, it is necessary to cultivate respect for medical workers, their hard and highly responsible work, and the healthcare system needs to guarantee patients high-quality and safe medical care [73]. By assuring patient satisfaction and strong relationships with them, the healthcare service providers can persuade the insurance companies to provide funding. Another way to attract payments is considered by the phenomenon of medical tourism, which forces medicine to develop rapidly by using modern technologies and improved patient care. “In some countries, the profitability of this business reaches 5% of the total volume and 15% of the revenue of the tourism business” [74]. One more approach is proposed when medical savings accounts are the property of every individual, which encourages citizens to take care of their health and increase responsibility for their own funds [75]. From the point of view of several authors, the funding of healthcare services from a private insurance could also be a good solution. The patient pays the provider for the healthcare services, and subsequently asks for the sum from the insurance company. Such funding alternatives are considered incentives for the organizations to provide only qualitative healthcare services that will lead to an effective system [76].

## 4. Conclusions

Nowadays, most countries act in the market economy, but a great part of them are trying to regulate high costs to make healthcare systems available and effective, such as the USA or Great Britain, Ireland, Denmark, Portugal, and Sweden. Usually, the healthcare systems are ruled by the government and managed by the public authorities due to its great importance for the population. The financing of the healthcare system has become an important issue, taking into account the scarcity of resources and the need to use funds in an effective and efficient manner. There is not a unique model, and the countries attract funds from a various range of sources, such as: national and local budgets, mandatory contributions of population, private insurances, patients’ funds, etc.

Based on a systematic analysis of the literature, including sources from the targeted countries, Russia and Romania, we tried to find answers to the research questions. As regards the first question, “Which financial model is better to follow for healthcare system?”, we can conclude that learning from other experiences is the best way to improve the financing of the healthcare systems. Using a hybrid model and a fair regulation can assure more funds that could be directed to improve the medical services delivered to the population. In the literature, it is highlighted that Russia and Romania are far behind the developed countries due to huge changes that took place in these countries during the end of last century, and beyond. These led them to keep ongoing and choose the new healthcare models for proper financing in market circumstances. Speaking of the transition to another healthcare model, scholars from Russia and Romania debated more or less the same issues about the financing models adopted by these countries. These models are considered with a low level of confidence, which have led to unstable results [12,39,40,43,52,54,59,61,77]. For the Russian situation, it is revealed that there is a lack of performing management and modern technologies used in healthcare services. Moreover, the lack of statistical data does not allow to fully assess the level of development of the healthcare model. For Romania, the situation is slightly different, as its officials try to find new sources of funding and improve the efficiency. One example is the implementation of measures to increase the resources available, such as raising taxes on cigarettes and pharmaceutical companies. However, despite these measures, Romania is still financed largely from compulsory social health insurance and state budget allocation.

The answers to the second question, “How to attract, accumulate and guide payments more effectively through healthcare system?”, are very diverse, but some central ideas emerge from the debates in the literature. Firstly, a public–private partnership is considered the most appropriate way to attract funds. The healthcare service providers should enter in competition for these funds, and as such, they will be forced to modernize their management and to use innovation to improve their services in order to become attractive both for patients and financing bodies [9,41,42,43,44,45]. Secondly, the customer relationships become very important, and the strategies of healthcare service providers should focus on assuring highly qualified staff who are able to achieve good professional performance, but also be able to communicate with patients in order to establish long-term relationships [46,47,48,49,50,51,52,53].

The results of this article could have an impact both for national regulation bodies, healthcare service providers, and academia. The findings in the specialty literature reveal that these three categories of entities should work together to find the best financing solutions, both by identifying new sources of funds and by increasing the efficiency and effectiveness of invested funds. The findings of this study also may help to guide the responsible actors from Russia and Romania to adapt government strategies and policies to the current challenges of the healthcare system.

### Limitations and Future Works

In the article, there are three limits: the number of concepts discovered in the reviewed research is rather small, and they are insufficient for merging Russian and Romanian practice in selecting a healthcare finance model; second, the study was constrained by the absence of recent research in the subject of selecting the best healthcare financial system, especially the influence of COVID-19 crisis; and third, there is a lack of experience in monitoring the healthcare financial system in the investigated countries.

Future research should focus on collecting information directly from the actors involved in the healthcare system, especially from service providers and patients. The new financing strategies considered after the lessons learned from the COVID-19 crisis should be also explored.

## Figures and Tables

**Table 1:** Inclusion and exclusion criteria.

Inclusion Criteria	Exclusion Criteria
It must have evaluation of at least one future result on considering financial models in healthcare, or detailed in conclusion;	Papers that claim the term “finances in healthcare” but are about someone’s particular business;
2. Paper has to be released between 2001 and 2021; 3. Article should contain information regarding finances for Romania and Russia.	2. Nonscientific articles; 3. Articles with a high risk of bias.

**Table 2:** Russian authors’ positions on the financial models.

Financial Model	Chubarova T.V.	Stiglitz J.E.	Sheiman I.M.	Shishkin S.V.	Chernenko E.M.	Lebedeva I.S.
Budgetary	+	+				
Social-Insurance			+	+		
Private					+	+

**Table 3:** Comparative characteristics of models of healthcare system in Russia.

System Model	Pros	Cons
Budgetary	Social priority; Focus on prevention; Highly qualified staff [46,52].	Lack of natural development of stimulant factors, slow growth in the quality of medical care, insufficient flexibility of organizational structures, long-term implementation of inefficient strategies; use of old medical technologies, restriction of the freedom to choose a medical institution [49].
Social-Insurance	Universality; Equality for receiving services; Equal accessibility [39].	Large share of costs financed by society and the state is taken up by the maintenance of disabled citizens [47]; Full funding is not possible at the expense of the state free medical care for the entire population of the country [43].
Private	Universality; Equality in receiving services; Equal accessibility [12].	Social guarantees of the need for residents to receive medical services are not provided; inability to implement municipal control, the possibility of crises of overproduction and incentives to supply unnecessary services [46].

**Table 4:** Romanian author’s positions on the choice of the most effective system.

Financial Model	Iacob AI.	Ianole R.	Oancea C.	Popescu C.	Besciu CD.
Budgetary		+		+	+
Social insurance			+		+
Private	+				+
Direct payments					+
Donors					+

**Table 5:** Comparative characteristics of healthcare financing models in Romania.

System Model	Pros	Cons
Budgetary	Mass character; Manageability [54].	The worst quality of medical care comparing to other, low stimulation, fewer flexibility of organizational structures, long-term implementation of belated strategies [54].
Social-Insurance	Different arrangements; Dependable; Availability [59].	Full allocation of funds is unrealistic due to state free medical support for all residents [59].
Private	Rise to a competitive market; Increase quality; Relation doctor–patient [61].	Extra increase in household costs; social guarantees of the need for residents to receive medical services are not provided; inability to implement municipal control [61].
Direct Payments	Better allocation of investments; Transparency of payments [40].	Looks like an imposed service to a patient and needs more burnishing within new technologies [40].
Donors	No limits per one payment; Gets more acceptance from patient [62].	Needs advertisement package. Payments are very rare. This system cannot work by its own and solely [62].

**Figure A1:**
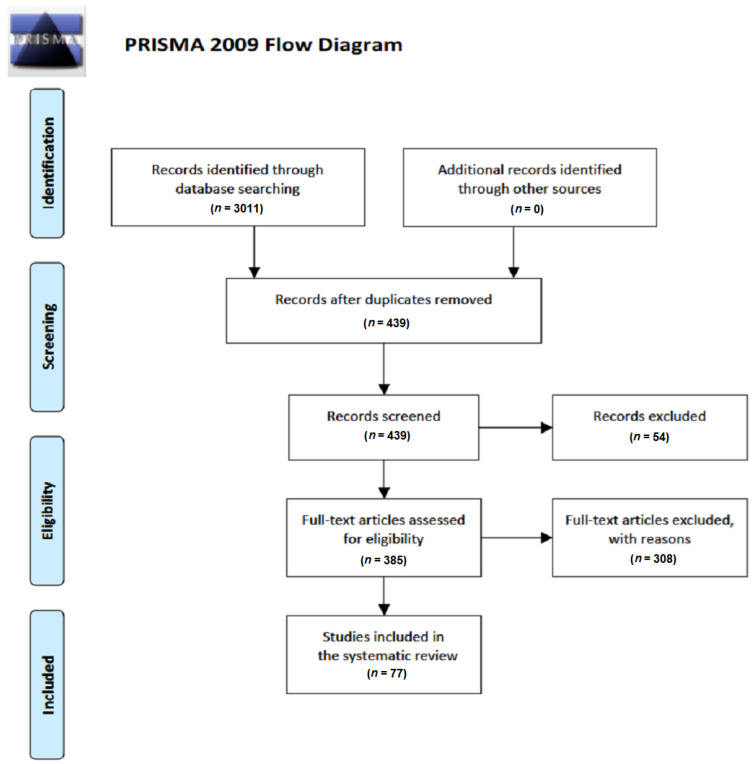
PRISMA Flow Diagram.

## Data Availability

Not applicable.
